# Prevalence and predictors of proton pump inhibitor partial response in gastroesophageal reflux disease in systemic sclerosis: a prospective study

**DOI:** 10.1038/s41598-020-57636-0

**Published:** 2020-01-21

**Authors:** Chingching Foocharoen, Kitti Chunlertrith, Pisaln Mairiang, Ajanee Mahakkanukrauh, Siraphop Suwannaroj, Suwassa Namvijit, Orathai Wantha, Ratanavadee Nanagara

**Affiliations:** 1 Division of Allergy-Immunology-Rheumatology, Department of Medicine, Khon Kaen University, Khon Kaen, 40002 Thailand; 2 Division of Gastroenterology, Department of Medicine, Khon Kaen University, Khon Kaen, 40002 Thailand; 3 Pharmacy Department, Khon Kaen University, Khon Kaen, 40002 Thailand; 4 Division of Nursing, Faculty of Medicine, Khon Kaen University, Khon Kaen, 40002 Thailand

**Keywords:** Systemic sclerosis, Gastro-oesophageal reflux disease

## Abstract

Proton pump inhibitor (PPI) twice daily dosing is a standard therapy for gastroesophageal reflux disease (GERD) in systemic sclerosis (SSc) but there is no data on its response rate or the predictors of PPI-partial response GERD. Aims were to determine the prevalence of PPI-partial response GERD in SSc and to define its predictors. A prospective study was conducted in SSc patients with GERD. The patients were treated with omeprazole 20 mg bid for 4 weeks. The severity of symptom-grading by visual analogue scale (VAS) and frequency of symptoms by frequency scale for symptoms of GERD (FSSG) were assessed at baseline and 4 weeks after treatment. PPI-partial response GERD was defined as less than 50% improvement in the VAS for severity of symptom as well as acid reflux score by FSSG after treatment. According to the sample size calculation, 243 SSc-GERD patients were enrolled; of whom 166 (68.3%) had the diffuse cutaneous SSc. PPI-partial response GERD was found in 131 SSc patients (prevalence 53.9%; 95%CI 47.4–60.3). The multivariate analysis revealed that esophageal dysphagia was an only predictor the PPI-partial response GERD (OR 1.82; 95%CI 1.01–3.29) while neither SSc subset nor severity of skin tightness were significantly associated with PPI-partial response GERD. Half of the SSc patients were PPI-partial response GERD. Esophageal dysphagia was the only predictor of PPI-partial response GERD in SSc patients. Screening for dysphagia before starting GERD treatment is helpful for assessment the risk of PPI refractoriness GERD in SSc patients.

## Introduction

Systemic sclerosis (SSc) is a systemic connective tissue disease that causes skin thickness and collagen deposition in internal organs. The pathogenesis of SSc is not well understood. Inflammation seems not to predominate in SSc which is different from other connective tissue diseases.

Gastrointestinal tract involvement has been reported in SSc—with a prevalence between 54 and 90%: this involvement trends to high morbidity^1,2^. The most frequent complication of SSc involves the esophagus (range, 30–96% of cases)^2–5^. Gastroesophageal reflux disease (GERD) is a clinical presentation of esophageal involvement in both the diffuse cutaneous SSc (dcSSc) and limited cutaneous SSc (lcSSc) subsets. Dysphagia, heart burn, and regurgitation are the common presentations of GERD. Chronic cough, recurrent pneumonia, laryngitis, and/or laryngospasm can present as extra-esophageal symptoms of GERD^3,6,7^. Interstitial lung disease has been reported to occur with GERD in SSc^8^; however, the pathophysiology is uncertain. Excessive deposition of collagen in the lamina propria and muscularis mucosae leads to hypotony of the lower esophageal sphincter which may develop into GERD in SSc^1^. Recent data indicate that there is no significant association between the extent of esophageal damage and the intensity of symptoms, duration of disease, or SSc subset^9^.

Typically, a diagnostic test is not required for GERD assuming there are the typical manifestations for GERD and the patient responds to therapy^10,11^. Endoscopy helps to differentiate whether the esophagitis is associated with GERD or is a therapy failure^12^. A physiological test—such as 24 h pH monitoring—could be used to confirm the presence of abnormal acid exposure of the esophagus in the event there are no esophageal lesions despite otherwise typical reflux symptoms^12,13^. Although acid reflux is a typical finding of GERD, non-acid reflux can occur in GERD. The characteristics of the reflux component were studied and categorized into three groups: acid, weakly acid, or weakly alkaline. An attendant drop in esophageal pH to less than 4 fulfilled the definition of acid reflux while an esophageal pH of 7 was the cut-off for weakly acid or weakly alkaline reflux^14^. Impedance plus pH monitoring are indicative of increased sensitivity for both acid and non-acid reflux in GERD^14^.

The GERD Questionnaire (GERD-Q) provides a non-invasive screening tool with high sensitivity for diagnosis of gastroesophageal reflux disease in systemic sclerosis^15^. A GERD-Q score of ≥4 and ≥8 indicated a respective sensitivity and specificity of 96.9% and 50%, and 65% and 100% for diagnosis GERD in SSc^15^. GERD-Q could be used to diagnose GERD in SSc; particularly when endoscopy cannot be performed (i.e., mouth opening is limited and/or 24 h pH monitoring is not available).

Modifications in lifestyle—including acidic food avoidance, weight reduction, smoking cessation, alcohol drinking reduction, small and frequent meals consumption, and eating more than 3 h before bedtime—are well-known, non-pharmacological treatments for GERD^12,16^. Daily administration of a proton pump inhibitor (PPI) are effective GERD therapies (i.e., 20 mg esomeprazole, 40 mg pantoprazole, 30 mg lansoprazole, or 40 mg omeprazole). The duration of treatment is 4–8 weeks albeit the evidence indicates that the response to treatment is not different between 4 and 8 weeks^13^. Prolonging PPI therapy to 8 weeks was not associated with any increased response even though it reduced relapse over against 4 weeks^17^.

PPI resistance has been reported in both erosive and non-erosive esophagitis GERD. The following were likely associated with PPI resistance—particularly non-erosive GERD: female, underweight, esophageal hiatal hernia, *Helicobacter pylori* infection, smoking, and non-acid reflux^18–20^. The rate of complete response increases by increasing the dose of PPI^19^, by adding prokinetics or by adding to an anti-anxiety drug^21,22^.

An effective therapy for uncomplicated GERD is a twice daily dose of PPI albeit there is no published research on the twice daily dose of PPI or the prevalence of PPI non-responsive or partial responsive GERD in SSc. The predictor of PPI-partial response GERD and the strategy for treatment in SSc with PPI-partial response GERD have yet to be investigated. We sought to find out the prevalence of SSc with PPI-partial response GERD.

## Method

A prospective clinical trial was performed at the Scleroderma Clinic, Srinagarind Hosptial, Khon Kaen University, Khon Kaen, Thailand. The trial featured a 4-week, open-label protocol. All eligible SSc patients clinically diagnosed as GERD were treated with omeprazole as per the standard protocol. The study was conducted between May 2013 and May 2018.

We enrolled the SSc patients age 18–65 years who had clinically GERD but not taking any prokinetic drug or PPI within 2 weeks prior to the enrollment. The patients who (a) were breast feeding or pregnant, (b) had a prior history of surgical procedure or therapeutic endoscopy owing to severe erosive esophagitis, (c) presented with Barrett esophagus, (d) were disable or not able to do daily activity, (e) indicated of active neoplastic disease, (f) presented uncontrollable severe medical disorders (i.e., airway disease, heart, renal or liver disease), (g) had current infection needing systemic antimicrobial agent, (h) had a history of omeprazole hypersensitivity, (i) received prohibited concomitants that might attenuate or affect GERD symptoms (i.e., oral bisphosphonate, ferrous salt, digoxin, tetracycline, or isoniacid) were excluded.

### Baseline assessment

All eligible patients were assessed at baseline, for medical history, frequency of symptoms using frequency scale for the symptoms of GERD (FSSG), symptoms severity using a visual analogue scale (VAS), and quality of life using EQ-5D score.

### Intervention

All eligible subjects received omeprazole 20 mg twice daily 30 minutes before meal for 4 weeks: a total of 56 capsules as a standard therapy. The medical treatments for SSc and concomitants—aside from prohibit medications—were given at the discretion of the attending physician.

***Primary endpoint***: prevalence of PPI-partial response GERD after omeprazole therapy for 4 weeks. ***Secondary endpoint***: (a) predictors associated with PPI-partial response GERD (b) quality of life of SSc-related GERD evaluated by EQ-5D (c) changing frequency of GERD symptoms assessed by FSSG^23^ and (d) changing severity of regurgitation and heart burn evaluated by visual analogue score (VAS) compared to baseline data.

### Information of study drug

Omeprazole (Miracid) a product by Berlin Pharmaceutical Industry Co., Ltd. Bangkok, is a proton pump inhibitor in form of a delayed-release capsule containing 20 mg of omeprazole/capsule. Side effects are rare but can include skin rash, headache, dizziness, back pain, and gastrointestinal discomfort. The patients with any history of hypersensitivity to benzimidazoles is contraindicated for prescription.

### Operational definitions

Systemic sclerosis (SSc) is diagnosed by using the American College of Rheumatology criteria^24^. SSc is divided as either the limited cutaneous SSc (lcSSc) or diffuse cutaneous SSc (dcSSc) per LeRoy *et al*.^25^.

The definition of GERD is fulfilled when the subject complains of regurgitation and/or heartburn and has a GERD-Q score greater than 8^15^. Heartburn is characterized as the burning sensation or discomfort back of the sternum, radiating to the neck and worsening after taking foods or upon lying down, and improving with antacids ingestion^26^. Regurgitation is the feeling of flow of gastric contents reflux into the mouth or hypopharynx^26^.

The PPI-partial response GERD is defined when the severity of reflux symptoms assessed by VAS and the frequency of GERDs symptoms evaluated by FSSG improve by less than 50% 4 weeks after omeprazole treatment compared to baseline data.

The onset of SSc was the date when the patients had his/her first symptoms of scleroderma. Duration of SSc was calculated from the date of patient enrollment to the date of the first non-Raynaud symptoms of SSc. Duration of GERD after onset of the disease was calculated by subtraction of the onset date of having GERD symptom and the onset of SSc. Raynaud’s phenomenon was a type of peripheral vasospasm and it was defined by a changing of skin color to white or blue at periphery (finger, nose or ears) because of blood flow reduction. The modified Rodnan skin score (mRSS) is used as a skin thickness assessment which assesses 17 areas of skin, including the face, anterior chest, abdomen, both arms, both forearms, both hands, fingers, both thighs, both legs and both feet. Each area has 4 scores (0–3) depends on the severity of skin thickness. The respective score 0, 1, 2, and 3 is normal skin, mild thickness, moderate thickness, and severe thickness. The mRSS is a summation of the score from all 17 skin assessment areas (range, 0–51). The pulmonary fibrosis is defined when fibrosis was detected by either chest radiographic or high resolution computed tomography (HRCT). Pulmonary arterial hypertension (PAH) was defined by a mean pulmonary arterial pressure ≥25 mmHg and a pulmonary capillary wedge pressure <15 mmHg from right heart catheterization^27^. Anemia was confirmed when Hb <12.0 g/dL in females and <13.0 g/dL in males. Low dose aspirin is defined by aspirin in the dosage of 81–100 mg/d. Steroid using is included any dose of steroid used. Immunosuppressant is included any immunosuppressive drugs as the following; cyclophosphamide, methotrexate, azathioprine and mycophenolate mofetil.

### Sample size

The sample size calculation was applied by the previous prevalence of PPI resistance from the literature review in which the prevalence of SSc in the general population was 1:100,000 and the previous prevalence of PPI resistance was 20%^28^. In order to detect a difference at 95% precision and alpha error of 0.05, the sample size was 246. We thus included 250 patients in the study.

### Statistical analysis

Patient baseline characteristics were summarized using descriptive statistics (i.e., percentages, means, and standard deviations). Comparisons were done using Student’s unpaired *t* test or the Man-Whitney U test where appropriate. The respective prevalence of PPI-partial response GERD with the 95% confidence interval (CI) was calculated. The odds ratio with 95%CI was used to assess which clinical characteristics predicted PPI-partial response GERD. Statistically significant variables (with a P < 0.1) were entered into a multivariate logistic regression model. All p values were two-tailed, and a p < 0.05 was required for statistical significance. All statistics were done using STATA version 11.2 (Stata Corp. College Station, TX, USA).

The Human Research Ethics Committee of Khon Kaen University approved the study as per the Helsinki Declaration and the Good Clinical Practice Guidelines (HE561044). All eligible patients signed informed consent before enrollment. The sponsor had no role in the study. The trial registration number NCT03561233.

### Compliance with ethical standards

#### Research involving human participants

Ethical approval: All procedures performed in studies involving human participants were in accordance with the ethical standards of the institutional and/or national research committee and with the 1964 Helsinki declaration and its later amendments or comparable ethical standards. The Human Research Ethics Committee of Khon Kaen University approved the study as per the Helsinki Declaration and the Good Clinical Practice Guidelines (HE561044).

#### Informed consent

Informed consent was obtained from all individual participants included in the study.

### Consent for publication

All of authors consent for publication and grant the Publisher exclusive license of the full copyright.

## Results

**Figure 1:**
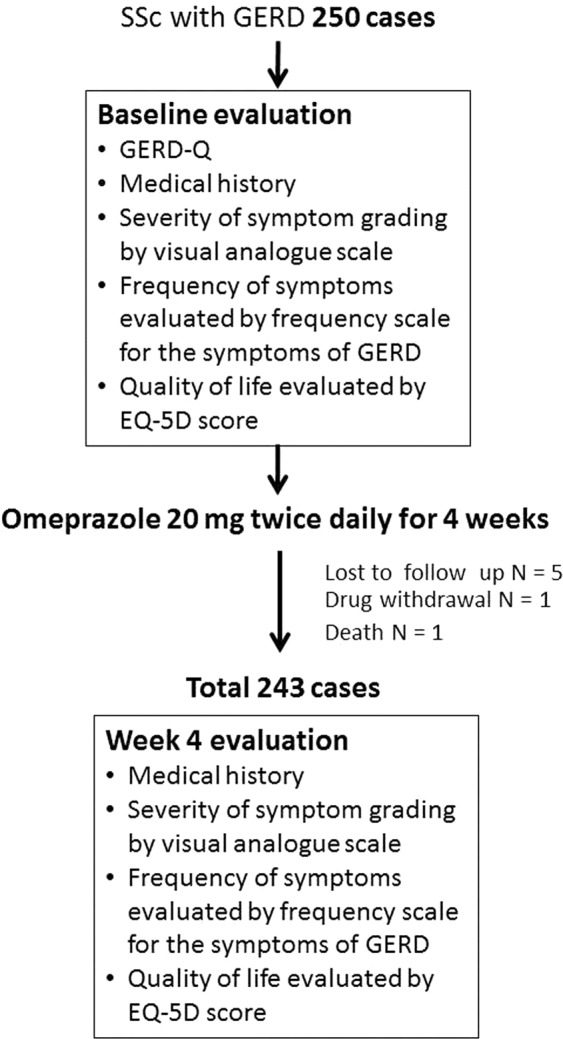
Flow chart of the study.

A total of 250 SSc patients diagnosed GERD were recruited to the study of whom 5 were lost to follow up, one had drug withdrawal and death and one died suddenly. A total of 243 SSc patients with GERD completed the follow up and were included in the analysis. The flow chart of the study is presented in Fig. 1.

The female to male ratio was 1.8:1 (157 to 86). The majority of patients had the diffuse SSc subset (dcSSc) (166 cases; 68.3%). The mean age was 55.0 ± 9.8 years (range, 25.5–80.0). The respective median duration of disease and duration of GERD after onset of SSc was 3.1 years (interquartile range; IQR 1.0–7.7) and 1.8 years (IQR 0.2–5.7).

PPI-partial response GERD was defined in 131 SSc patients with GERD with a prevalence of 53.9% (95%CI 47.4–60.3). In the univariate analysis, esophageal dysphagia, high baseline dysmotility score by FSSG, and high baseline severity of regurgitation by VAS were associated with PPI-partial response GERD (Table 1). While old age, duration of disease, SSc subset, skin tightness severity and concomitant low dose aspirin, steroid, and immunosuppressant using were not associated with the response to PPI in SSc with GERD (Table 1). In the multivariate analysis, only esophageal dysphagia was a significant predictor of PPI-partial response GERD in SSc patients (OR 1.82 (95%CI 1.01–3.29)) (Table 2).

**Table 1:** Univariate analysis of the predictors of PPI-partial response GERD in SSc.

Clinical characteristic	PPI Response GERD N = 112 (%)	PPI-PR GERD N = 131 (%)	Odds Ratio (95%CI)	p-value
Female	74 (66.1)	83 (63.4)	0.89 (0.51–1.56)	0.66
Age >60 years	37 (33.0)	35 (26.7)	0.74 (0.41–1.33)	0.28
Age; years: mean ± SD	55.3 ± 10.2	54.8 ± 9.5	—	0.72
dcSSc subset	73 (65.2)	93 (71)	1.31 (0.73–2.33)	0.33
BMI <18.5 kg/m^2^	20 (17.9)	28 (21.4)	1.25 (0.63–2.51)	0.49
Duration of disease >5 years	47 (42.0)	45 (34.4)	0.72 (0.42–1.26)	0.22
Duration of disease; years: median (IQR)	3.4 (1.1–8.0)	3.1 (1.0–6.6)	—	0.58
Duration of GERD; years: median (IQR)	1.83 (0.17–4.66)	1.84 (0.17–5.0)	—	0.64
**Clinical characteristics**				
WHO functional class > I	46 (41.1)	63 (48.1)	1.24 (0.71–2.16)	0.42
Raynaud’s phenomenon	38 (33.9)	59 (45.0)	1.60 (0.92–2.78)	0.08
Digital ulcer	18 (16.1)	25 (19.1)	1.23 (0.60–2.56)	0.54
Gangrene	1 (0.9)	4 (3.1)	3.50 (0.34–173.67)	0.24
Vasculopathy	47 (42.0)	69 (52.7)	1.54 (0.90–2.64)	0.09
Telangiectasia	30 (26.8)	44 (33.6)	1.38 (0.77–2.50)	0.25
Calcinosis cutis	3 (2.7)	0	NA	
Salt and pepper skin	45 (40.2)	61 (46.6)	1.30 (0.75–2.23)	0.32
Edematous skin	16 (14.3)	30 (22.9)	1.78 (0.87–3.72)	0.09
Tendon friction rub	3 (2.7)	8 (6.1)	2.36 (0.55–14.12)	0.20
Hand deformity	53 (47.3)	58 (44.3)	0.88 (0.52–1.51)	0.63
Synovitis	5 (4.5)	6 (4.6)	1.02 (0.25–4.38)	0.97
Muscle weakness	5 (4.5)	9 (6.9)	1.58 (0.46–6.18)	0.42
Dysphagia	39 (34.8)	74 (56.5)	2.43 (1.40–4.23)*	<0.001*
Stomach symptom	42 (37.5)	53 (40.5)	1.13 (0.65–1.97)	0.64
Constipation	12 (10.7)	20 (15.3)	1.50 (0.66–3.55)	0.30
Diarrhea	1 (0.9)	3 (2.3)	2.60 (0.50–137.77)	0.39
Pulmonary fibrosis	41 (36.6)	58 (42.3)	1.37 (0.80–2.39)	0.23
Pulmonary arterial hypertension	5 (4.5)	7 (5.3)	1.21 (0.32–4.97)	0.75
Night cough	52 (46.4)	67 (51.2)	1.19 (0.64–2.22)	0.55
Weight loss	17 (15.2)	21 (16.0)	1.07 (0.50–2.29)	0.86
mRSS > 20 points	12 (10.9)	20 (20.8)	2.14 (0.98–4.90)	0.06
**Laboratory findings**				
Anemia^a^	55 (49.6)	63 (48.8)	0.97 (0.57–1.67)	0.91
Low serum albumin level^b^	2 (2.3)	3 (3.1)	1.37 (0.15–16.77)	0.73
hyperCKaemia^c^	27 (24.1)	25 (19.1)	0.74 (0.38–1.44)	0.34
Anti-topoisomerase I antibody positive	79 of 102 (77.5)	96 of 111 (86.5)	1.86 (0.86–4.11)	0.09
**Coexisting disease**				
Metabolic syndrome	9 (8.0)	14 (11.5)	1.48 (0.58–4.00)	0.37
Thyroid disease	0	6 (4.6)	NA	—
Viral hepatitis	4 (3.6)	1 (0.8)	0.21 (0.01–2.15)	0.12
**Patients behavior**				
Time interval to go to sleep after meal < 2 h	39 (35.1)	40 (30.5)	0.81 (0.46–1.44)	0.45
Alcohol drinking	4 (3.6)	3 (2.3)	0.63 (0.09–3.84)	0.55
Coffee drinking	29 (25.9)	32 (24.4)	0.93 (0.50–1.73)	0.79
Smoking	4 (3.6)	5 (3.8)	1.07 (0.22–5.54)	0.92
**Concomitant medication**				
Low dose aspirin	101 (90.2)	119 (90.8)	1.08 (0.41–2.80)	0.86
Prednisolone	63 (56.3)	81 (61.8)	1.26 (0.73–2.17)	0.38
Immunosuppressant	36 (32.1)	43 (32.8)	1.03 (0.58–1.83)	0.91
Baseline acid reflux score by FSSG: median (IQR)	9 (6.5–12)	10 (7–14)	—	0.23
Baseline dysmotility score by FSSG: median (IQR)	7 (5–9)	8 (6–11)	—	0.02*
Baseline severity of heartburn by VAS: median (IQR)	50 (29–66)	50 (22–65)	—	0.65
Baseline severity of regurgitation by VAS: median (IQR)	44 (23–70)	53 (30–80)	—	0.048*

*Statistically significant p < 0.05.

^a^Hb < 12 g/dl in female, <13 g/dl in male, ^b^<3.0 gm/ml, ^c^CK > 200 IU/L.

PPI proton pump inhibitor, PPI-PR GERD proton pump inhibitor partial response gastroesophagel reflux disease, dcSSc diffuse cutaneous systemic sclerosis, BMI body mass index, WHO functional class World Health Organization functional class, IQR interquartile range, mRSS modified Rodnan skin score, FSSG frequency scale for symptoms of GERD, VAS visual analogue scale.

**Table 2:** Multivariate analysis of the predictors of PPI-partial response GERD in SSc.

Clinical characteristic	Crude Odds Ratio (95%CI)	Adjusted Odds Ratio (95%CI)	p-value
Raynaud’s phenomenon	1.60 (0.92–2.78)	1.26 (0.70–8.30)	0.44
Edematous skin	1.78 (0.87–3.72)	1.59 (0.73–3.44)	0.24
Dysphagia	2.43 (1.40–4.23)	1.82 (1.01–3.29)	0.04*
mRSS >20	2.14 (0.98–4.90)	1.76 (0.78–3.98)	0.17
Anti-topoisomerase I antibody positive	1.86 (0.86–4.11)	1.70 (0.79–3.66)	0.17
Baseline dysmotility score by FSSG every 1 point	—	1.04 (0.95–1.14)	0.40
Baseline severity of regurgitation by VAS every 1 scale	—	1.00 (0.99–1.01)	0.45

*Statistically significant p < 0.05.

mRSS modified Rodnan skin score, FSSG frequency scale for symptoms of GERD, VAS visual analogue scale.

**Figure 2:**
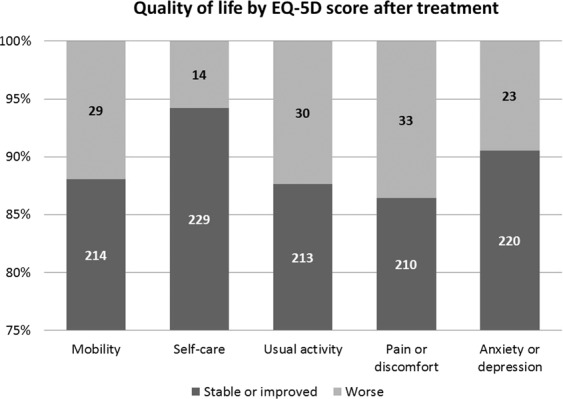
Quality of life by EQ-5D score after treatment.

**Figure 3:**
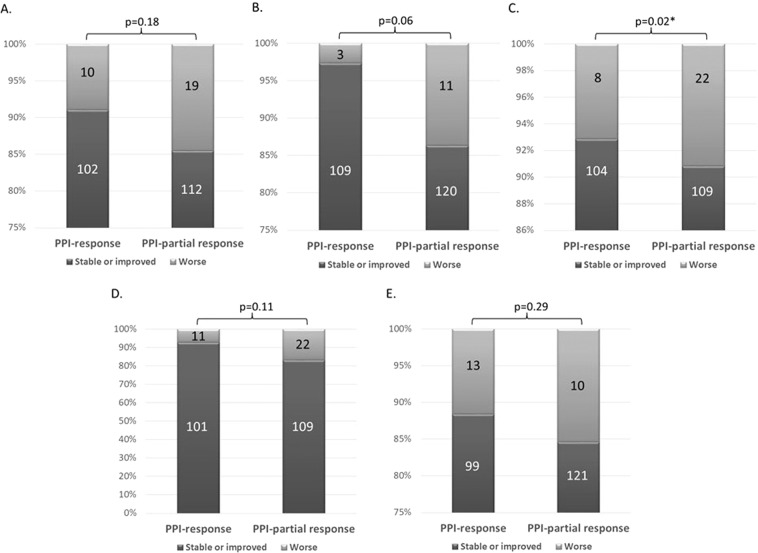
Changing quality of life classified by clinical response to PPI (**A**). Mobility, (**B**). Self-care, (**C**). Usual activity, (**D**). Pain or discomfort, (**E**). Anxiety or depression.

The majority of the patients had stable or improved quality of life as evaluated per the EQ-5D score after taking PPI therapy: 88% (214 cases) in mobility; 94% (229 cases) in self-care; 87.7% (213 cases) in usual activities; 86.4% (210 cases) in pain/discomfort; and, 90.5% (220 cases) in anxiety/depression (Fig. 2.) When comparing each module, the quality of life between the SSc patients with a PPI response and those with a PPI-partial response GERD, the patients with a PPI-partial response GERD experienced significant worsening of usual activity than those who had PPI response GERD (p = 0.02). Other modalities with respect to quality of life showed no significant difference (Fig. 3).

An adverse drug reaction was observed in 3 patients; one fatigue, one leg pain, and one upper respiratory tract infection: none needed hospitalization or any additional treatment.

## Discussion

PPI is a standard therapy for GERD in the general population, including for patients with SSc. The response rate of PPI therapy in GERD in non-SSc patients was around 22–59%^29–34^. The response rate varies according to differences in definitions, population, medication, and duration of treatment. Previous studies revealed that non-SSc patients who received a single daily PPI dose had a lower response rate than those who received a twice daily dose. In addition, those who had non-erosive esophagitis had a lower response rate than those who had erosive esophagitis^30,34,35^.

The response rate of GERD treatment among our SSc patients (by definition PPI-partial response GERD) was less than 50% improvement vis-à-vis severity of heartburn and frequency of heartburn after taking omeprazole 20 mg twice daily for 4 weeks, but that is higher than among non-SSc patients even with high dose PPI therapy. Unfortunately, we did not have control or placebo group, so we cannot provide the clinical GERD improvement in the patients who receive only lifestyle modification or placebo treatment. Although the high rate of the PPI-partial response GERD in SSc patients, most of the patients had stable or improved quality of life in 5 dimensions of health state that included mobility, self-care, usual activity, pain/discomfort and anxiety/depression. The result reflects that GERD is troublesome for the patients’ quality of life and PPI still has a benefit for the patient despite of PPI-partial response GERD. We, therefore, suggest PPI as the first line of treatment for GERD in SSc patients.

According to the pathophysiology of the disease, motility problems constituted the mechanism of GERD development in SSc^36^. Esophageal dysmotility could thus be a factor in the refractoriness of SSc patients with GERD to PPI treatment. The reason is supported by our analysis that dysphagia—which is a symptom of esophageal dysmotility—is a predictor of PPI-partial response GERD.

The dysphagia is a well-known classical symptom of esophageal involvement in SSc. The involvement can lead to difficulty clearing food, causing reflux symptoms (i.e., heart burn and regurgitation)^37^. It is thus not surprising that our result revealed that dysphagia is a strongly predictor of PPI-partial response GERD in the SSc patients.

Although esophageal dysmotility is the suspected mechanism of GERD in SSc, the exact pathophysiology of GERD in SSc is unknown and it is debated whether the pathogenesis of GERD in SSc is different from the general population^38^. Alternatively, the esophageal dysmotility in SSc could be caused by (a) vasculopathy leading to local esophageal ischemia and esophageal dysfunction^37^, (b) neuropathy of nerves supplying the esophagus, and (c) fibrosis of the smooth muscle of the esophagus and/or lower esophageal sphincter^39^. PPI acts as an acid suppression so it can relieve heart burn, which is the typical presentation of GERD; however, it has no effect on esophageal motility and/or lower esophageal sphincter pressure, which are the possible mechanism of GERD in SSc. So, esophageal dysmotility might explain why the prevalence of PPI-partial response GERD in SSc is higher than in non-SSc patients. Due to budgetary limitations, we did not perform esophageal manometry which is a functional assessment tool of the esophagus, so we cannot evaluate how many patients had esophageal dysmotility and how severe of esophageal dysmotility in our SSc patients is.

Prokinetic agents has a mechanism of action on the esophageal motility by facilitating esophageal motility and increasing lower esophageal sphincter pressure^40^. It can thus be helpful for relief of GERD symptoms in patients who have failed PPI treatment. A recent randomized control trial provided strong evidence that a twice daily dose of PPI (omeprazole) in combination with domperidone—a prokinetic agent—10 mg TID has high efficacy for relief GERD symptoms and increases the response rate of treatment in SSc patients who were non-responsive to PPI treatment^41^. Based on our results, and other reports, we suggest screening for any history of dysphagia before starting GERD treatment for all SSc patients who have clinically suspected GERD in order to assess the risk of PPI-partial response GERD. Prokinetic drug PPI add-on therapy should be considered in the SSc patients who have coexisting dysphagia and GERD. The early combination therapy might improve GERD symptom and PPI response rate in such patients.

The other possible explanation of the high rate of PPI-partial response in our patients could be related to the bioavailability of PPI. Drug bioavailability—particularly drug metabolism—can be affected by genetic variation. A previous study showed that Asian populations had a higher rapid metabolism than Caucasian populations^34^. The genotype expressed cytochrome P450 2 C enzyme is supposed to be involved in the ability to metabolize PPI^34^. The genetic variation might be a factor that influences the PPI response rate in our patients. There have been no pharmacogenetics study on PPI metabolism in SSc patients; we are now doing a further investigation on the genetic differences between SSc patients defined as PPI-partial response GERD and PPI response GERD.

The limitations of the study follow: (a) endoscopy or 24 h pH monitoring for diagnosis of GERD was not performed in our patients because it is an invasive procedures so we instead used the GERD-Q which has demonstrated high specificity for diagnosis of GERD^15^; (b) the type of GERD (erosive and non-erosive esophagitis) could not be defined nor the response rate for each type; (c) there were several under-controlled confounders that could have influenced the GERD symptoms and outcomes of treatment with PPI such as food, beverage, lifestyle, and stress. It was uncertain that all of our patients did according to our suggestions (lifestyle modification) even though monitoring was performed by asking patient lifestyle questions before and at the end of the study; (d) we included any drinks of alcohol/coffee and any packs of smoke into the analysis, we therefore cannot investigate the association between the quantity of the drink/pack of smoke and the PPI response GERD in SSc; (e) we did not use the definition of SSc according to ACR/EULAR 2013 classification criteria because most of the patients were enrolled before the classification criteria was released; and (f) we did not use PPI other than omeprazole or having a control group, we therefore cannot provide the prevalence of PPI-partial response GERD of other PPI drugs or placebo.

The strengths of the study were (a) the number of patients in the study was sufficient for determining the prevalence and the predictor of PPI-partial response standard dose therapy in SSc with GERD; (b) patient behaviors associated with GERD were included (i.e., smoking, alcohol drinking, time interval to go to bed), so could differentiate the possible cause of PPI-partial response GERD between the disease and patient behaviors; (c) the study included quality of life as an outcome; and, (d) the results assessed the feasibility of GERD treatment in SSc. Our study is fundamental for better care of GERD in SSc patients.

## Conclusion

Half of the SSc patients were PPI-partial response GERD. Esophageal dysphagia was the only predictor of PPI-partial response GERD in SSc patients. Screening for dysphagia before starting GERD treatment is helpful for assessment the risk of PPI refractory GERD in SSc patients.

## Data Availability

There is no data and material available.
